# Intersection of Two Checkpoints: Could Inhibiting the DNA Damage Response Checkpoint Rescue Immune Checkpoint-Refractory Cancer?

**DOI:** 10.3390/cancers13143415

**Published:** 2021-07-08

**Authors:** Peter H. Goff, Rashmi Bhakuni, Thomas Pulliam, Jung Hyun Lee, Evan T. Hall, Paul Nghiem

**Affiliations:** 1 Department of Radiation Oncology, University of Washington, Seattle, WA 98195, USA; goffp01@uw.edu; 2 Division of Dermatology, Department of Medicine, University of Washington, Seattle, WA 98109, USA; rbhakuni@uw.edu (R.B.); thpullia@uw.edu (T.P.); jlee24@uw.edu (J.H.L.); 3 Institute for Stem Cell and Regenerative Medicine, Department of Bioengineering, University of Washington, Seattle, WA 98109, USA; 4 Division of Medical Oncology, Department of Medicine, University of Washington, Seattle, WA 98109, USA; evanh@uw.edu; 5 Clinical Research Division, Fred Hutchinson Cancer Research Center, Seattle, WA 98109, USA

**Keywords:** immune checkpoint inhibitors, ATR, ATM, DNA-PK, DNA damage response inhibitors, Merkel cell carcinoma, PD-1 pathway, cell cycle checkpoint, immunogenic cell death

## Abstract

**Simple Summary:**

Immunotherapies have revolutionized the management of advanced stage cancer; however, not all patients benefit given many cancers are resistant to immune checkpoint inhibitors. To support rapid growth, malignant cells frequently bypass the cell cycle checkpoints that normally ensure high-fidelity DNA replication prior to cell division. Therefore, cancer cells (lacking early cell cycle checkpoints) are under high replication stress and rely more heavily on the DNA damage response (DDR) for survival and growth. DDR pathways are long-recognized anti-neoplastic targets. Recently, preclinical studies have demonstrated unexpected crosstalk between these pathways and the immune system. Here, we discuss emerging evidence linking cell cycle biology and the DDR to innate and adaptive immunity. We present a rationale for why Merkel cell carcinoma could serve as a paradigm for studying DDR inhibitors as novel agents to overcome resistance to programmed cell death-1 (PD-1) pathway blockade.

**Abstract:**

Metastatic cancers resistant to immunotherapy require novel management strategies. DNA damage response (DDR) proteins, including ATR (ataxia telangiectasia and Rad3-related), ATM (ataxia telangiectasia mutated) and DNA-PK (DNA-dependent protein kinase), have been promising therapeutic targets for decades. Specific, potent DDR inhibitors (DDRi) recently entered clinical trials. Surprisingly, preclinical studies have now indicated that DDRi may stimulate anti-tumor immunity to augment immunotherapy. The mechanisms governing how DDRi could promote anti-tumor immunity are not well understood; however, early evidence suggests that they can potentiate immunogenic cell death to recruit and activate antigen-presenting cells to prime an adaptive immune response. Merkel cell carcinoma (MCC) is well suited to test these concepts. It is inherently immunogenic as ~50% of patients with advanced MCC persistently benefit from immunotherapy, making MCC one of the most responsive solid tumors. As is typical of neuroendocrine cancers, dysfunction of p53 and Rb with upregulation of Myc leads to the very rapid growth of MCC. This suggests high replication stress and susceptibility to DDRi and DNA-damaging agents. Indeed, MCC tumors are particularly radiosensitive. Given its inherent immunogenicity, cell cycle checkpoint deficiencies and sensitivity to DNA damage, MCC may be ideal for testing whether targeting the intersection of the DDR checkpoint and the immune checkpoint could help patients with immunotherapy-refractory cancers.

## 1. Immunotherapy: Progress and Problems

Over the past decade, immune checkpoint inhibitors (ICIs) have progressed from early clinical trials to established pillars of treatment for many advanced solid malignancies [1]. There are now eight ICIs approved for a variety of indications which target programmed cell death-1 (PD-1) or its ligand, PD-L1, or cytotoxic T-lymphocyte-associated protein 4 (CTLA-4) [2,3,4]. Agents targeting PD-1 or PD-L1 (collectively “anti-PD-(L)1” therapies) have become dominant (seven of the eight approvals) in most settings given their higher efficacy and reduced toxicity relative to anti-CTLA-4 therapy [5,6,7]. Broadly speaking, these immune checkpoint pathways serve as negative regulators of T cell immunity. Their specific functions and patterns of cellular expression have been reviewed in detail [8,9]. The proportion of patients with advanced cancer who are eligible for ICI therapy increased from an estimated 1.5% in 2011 to 44% in 2018 [10], and increasing ICI utilization is associated with survival gains for patients with several advanced malignancies [11,12,13,14]. Despite the remarkable expansion in the number of agents and indications for ICIs in cancer therapy, response rates are affected by many factors and vary widely, as discussed below.

### 1.1. Clinical Efficacy of Immune Checkpoint Inhibitors

Response rates to ICIs vary widely by histology from essentially no benefit to greater than 50% in the most sensitive cancers [1]. Notably, many skin cancers are associated with a high tumor mutational burden (TMB) resulting from ultraviolet radiation (UV)-induced DNA damage [15,16]. Tumor neoantigens derived from DNA damage render them visible to anti-tumor T cells, and thus skin cancers are particularly sensitive to ICIs. The first FDA (United States Food and Drug Administration) approval granted for any ICI was for an anti-CTLA-4-targeting monoclonal antibody, ipilimumab, for melanoma in 2011 after showing a survival benefit in patients with pre-treated metastatic disease [17]. There have been many subsequent approvals for ICIs to treat skin cancers, and response rates for each of the following indications were in the impressive 30–60% range [18]. The majority of responses to ICIs are more durable than those achieved with chemotherapy or targeted therapies. Nivolumab (anti-PD-1) and pembrolizumab (anti-PD-1) were approved for melanoma in 2014 and 2015, respectively [19,20]. The combination of anti-PD-1 and anti-CTLA-4 agents was approved in 2015 for melanoma; overall survival at 5 years was 52% for ipilimumab/nivolumab vs. 44% for nivolumab alone [21]. Combining CTLA-4 and PD-1 inhibitors increases immune-related toxicities, although efforts are underway to optimize dosing to improve the tolerability of combined therapy [22]. Avelumab (anti-PD-L1) [23,24] and pembrolizumab [25,26] were approved for Merkel cell carcinoma (MCC) in 2017 and 2018, respectively. Cemiplimab (anti-PD-1) was approved for squamous cell carcinoma in 2018 [27] and for basal cell carcinoma [28] in 2021.

To increase response rates to ICIs across diverse cancer types, significant efforts have been made to identify biomarkers to inform patient selection. An increase in ICI responsiveness has been linked to a litany of factors that include tumor-intrinsic (e.g., high TMB [16] or PD-L1 expression [29]) and tumor-extrinsic factors (e.g., low neutrophil to lymphocyte ratio [30] and obesity [31,32]). Yet, no single factor or well-defined combination of factors has yielded a clinically reliable predictor of response to immunotherapy. Absent predictive markers, the typical approach is to empirically administer therapy to all eligible patients. As the average cancer patient treated with an ICI is not likely to have a meaningful response (although many histologies are highly responsive as above), some patients will experience significant toxicity without benefit. TMB can be readily determined and is one of the most clinically used, if imperfect, predictors of response to ICI [33]. Indeed, pembrolizumab was approved for treatment of all advanced solid tumors with a high TMB (≥10 mutations/megabase) on the basis of an overall response rate of 29% [34].

### 1.2. Limitations of Current Immune Checkpoint Inhibitors

With the urgent need to increase response rates to ICIs, tremendous efforts have been made and are ongoing to optimally combine them with existing or novel therapies. Some notable gains have been made in combining ICI with cytotoxic chemotherapy in lung [35] and esophageal cancers [36], for example. However, while chemotherapy reduces the systemic tumor burden, it also weakens the anti-tumor immune response (as lymphocytes are sensitive to cytotoxic agents), making this an approach that may not be suitable for all tumor types [37,38]. Combining radiation with ICI has been widely tested with some notable areas of success [39,40,41,42,43], although it may be immunosuppressive in some settings. Adding radiotherapy to immune therapy in ICI-resistant patients has generally not yielded a synergistic clinical benefit for unirradiated tumors despite very promising data in animal models and some early, promising clinical studies [39,44,45,46].

With new efforts to combine immune therapies to augment efficacy, immune related adverse events (irAEs) also become more prevalent. irAEs are immune responses induced by the relevant therapy against healthy tissues. The combination of immunotherapies has yielded some successes. Combining ipilimumab with anti-PD-1 agents in melanoma [21] or hepatocellular carcinoma (HCC) [47,48] may increase response rates modestly for resistant cancers but at the cost of a significant increase in the likelihood and severity of irAEs. Even for single-agent immunotherapy, irAEs are sometimes severe, may be irreversible and often lead to discontinuation of immunotherapy [49,50]. Despite the appeal of combing multiple immunotherapies to augment response rates, this approach will typically lead to greater irAE toxicities. One successful strategy to avoid augmented irAEs has been the use of intralesional immune-stimulating agents which typically do not increase systemic immune toxicity. Talimogene laherparepvec (T-VEC) is an oncolytic herpesvirus approved for intralesional melanoma injection [51]. T-VEC is well tolerated in combination with ICI and is being explored in immunotherapy-resistant disease [52,53]. 

### 1.3. New Approaches to Improve the Efficacy of ICI and Enhance Anti-Tumor Immunity

To expand the pool of patients benefitting from immunotherapy, efforts are underway to identify predictive biomarkers for ICI response in order to better select patients and inform future clinical trial design [54,55]. Novel therapies and combinations of existing treatments are also in development to aid patients with ICI-refractory disease. For example, the combination of ICI with molecularly targeted agents, such as the vascular endothelial growth factor receptor (VEGFR) inhibitor bevacizumab or multi-tyrosine kinase inhibitors (e.g., axitinib or cabozantinib), has shown promising efficacy in recent trials [56,57]. The development of agents targeting novel immune checkpoint proteins (e.g., lymphocyte activating 3 (Lag3), T cell immunoglobulin and mucin domain-containing protein 3 (Tim3)) is also well underway and could be combined with existing ICIs [58,59,60].

Identifying non-immune targets for anti-neoplastic drugs that may stimulate anti-tumor immunity, especially in the setting of ICI-refractory cancers, is particularly appealing because they are less likely to augment irAEs. Targets including cell cycle regulators (e.g., cyclin-dependent kinases or CDKs) and DNA damage response (DDR) proteins have long been of interest in cancer research. Clinically, the most advanced DNA damage repair-targeting agents are inhibitors of poly (ADP-ribose) polymerase (PARPi), which are actively being explored in combination with ICI [61,62,63]. PARPi have efficacy in BRCA-mutant (BRCA1/2 mutations) cancers deficient in homologous recombination (via “synthetic lethality”) but are currently of limited utility outside of this setting [64]. The status of PARPi has been extensively summarized and is not the focus of this review [65]. Instead, several new drug candidates targeting central regulators of the DDR are of emerging significance. These include ATR (ataxia telangiectasia and Rad3-related), ATM (ataxia telangiectasia mutated) and DNA-PK (DNA-dependent protein kinase) for which several inhibitors have entered clinical trials in the past several years [66,67]. Exciting preclinical data suggest these new agents have the potential to synergize with ICIs, potentially without increasing immune-related toxicity.

## 2. Crosstalk between the DNA Damage Response and Innate and Adaptive Immunity

Surgery, radiation and chemotherapy are the traditional three pillars of cancer therapy. The latter two selectively induce DNA damage in rapidly dividing tumor cells in contrast to healthy, normal tissues which divide less frequently. Accordingly, DDR pathways and cell cycle checkpoint biology have been intensely studied for decades with an eye toward developing molecularly targeted anti-cancer therapeutics.

The DNA damage response is a network of multiple interconnected pathways responsible for repairing damaged DNA and maintaining genomic integrity. The DDR pathways are integrated with cell cycle checkpoint proteins (e.g., Rb (retinoblastoma protein) and p53 (tumor protein p53)) such that when DNA damage is sensed, progression through the cell cycle is arrested until the damage may be repaired or, if irreparable, the cell may undergo regulated cell death to prevent the passage of potentially deleterious DNA mutations to daughter cells. There are three master regulatory proteins of the DDR: ATR, ATM and DNA-PK, which have been extensively reviewed [68]. These DDR proteins have some specificity for various DNA lesions with ATM and DNA-PK acting at double-strand DNA (dsDNA) breaks, while ATR is recruited to single-strand DNA (ssDNA) at stalled replication forks. These “transducer kinases” then act differentially at various points in the cell cycle [69].

Neoplastic cells frequently harbor mutations in cell cycle checkpoints, most commonly p53. Mutations in DDR proteins including functional deletions in ATM and DNA-PK proteins are also observed [70]. However, ATR, which acts in the S (DNA synthesis) and G2 (gap 2) phases of the cell cycle to ensure that DNA synthesis is complete prior to entering mitosis, is virtually never completely eliminated. Indeed, complete loss of ATR function has not been observed in humans to our knowledge, although its expression is greatly reduced in some cases of Seckel syndrome [71]. In contrast, ATM loss of function causes ataxia telangiectasia, which occurs at rates of up to ~1:40,000 individuals and is associated with a 20–30% lifetime cancer risk [72]. ATM heterozygosity is prevalent at rates of 1–2% in the general population and is a significant risk for the development of several malignancies, breast cancer in particular [72,73,74]. ATM mutations are present in approximately 5% of all cancers [75]. Human germline DNA-PK loss of function mutation is very rare but is a cause of severe combined immunodeficiency [76]. Mouse studies support ATR as a critical protein, with its knockout resulting in embryonic lethality [77], while ATM [78] and DNA-PK [79] knockout mouse models are viable. While ATM and DNA-PK expression may be lost in human cancer [70,75], ATR expression is generally maintained, although mutations and reduced expression levels are sometimes observed [80]. Dysregulation of DNA-PK leading to increased activity is also associated with aggressive cancers with poor outcomes [81,82]. Loss of cell cycle checkpoints supports the unrestrained growth characteristic of neoplastic cells. DDR protein deficiencies may benefit cancer cells by allowing them to bypass these cell cycle checkpoints and harbor a greater mutational burden. ATR’s role in ensuring completion of DNA synthesis, especially in the context of other cell cycle checkpoint or DDR pathway deficiencies, appears to be required in replicating cells, thus making it a particularly appealing therapeutic target.

Given the importance of cell cycle checkpoints and DDR biology in tumorigenesis, targeting DDR pathways and their effector proteins to halt cell cycle progression has been of great interest over the past two decades [83]. Inhibitors of ATR, ATM and DNA-PK have been studied in many different cancer types. They were initially explored for their abilities to sensitize cells to DNA-damaging agents, including radiation and cytotoxic chemotherapies, or to synergize with existing cell cycle checkpoint or DDR deficiencies (e.g., use of ATR inhibitors in ATM-deficient malignancies) to exert a direct cytotoxic effect [84,85]. The concept of synthetic lethality has perhaps been best demonstrated clinically with the use of PARPi in BRCA (breast cancer)-deficient cancers whereby PARP-mediated DNA damage repair becomes critical for maintaining genomic integrity with the loss of homologous recombination [86]. After more than a decade of delay between establishing that DDR proteins were desirable cancer therapy targets, potent and selective compounds eventually became available to target ATR, ATM and DNA-PK. Recent clinical studies have shown that several drug candidates appear to be tolerated in combination with radiation or a variety of chemotherapeutic agents, as discussed below.

**Figure 1:**
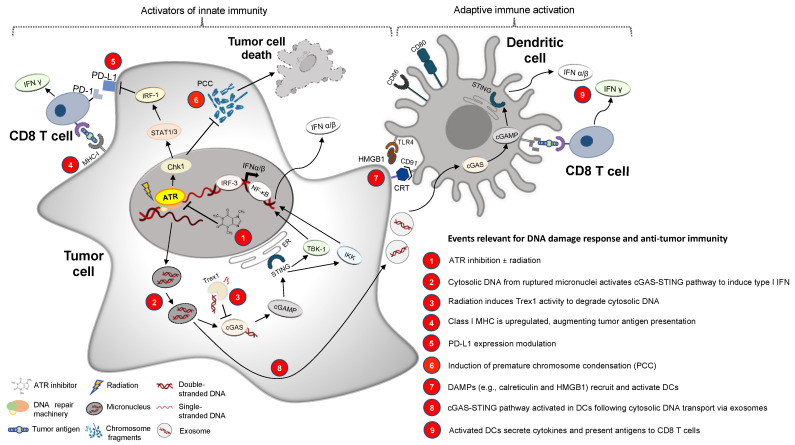
Events that relate targeted DDR (ATR) inhibition to anti-tumor immunity. Nine steps delineated in this figure summarize how ATR inhibition may augment innate immunity and reinvigorate adaptive immune re-sponses via cGAS-STING pathway activation, DAMP signaling, PD-L1 expression modulation, class I MHC upregula-tion and enhanced T cell priming via activation of dendritic cells (see text for explanation). Abbreviations: CD8/80/86/91: cluster of differentiation 8/80/86/91, cGAMP: cyclic guanosine monophosphate–adenosine monophosphate, cGAS: cy-clic GMP-AMP synthase, Chk1: checkpoint kinase 1, CRT: calreticulin, DAMPs: damage-associated molecular patterns, HMGB1: high mobility group box protein 1, IFN: interferon, IKK: IκB kinase, IRF-1 or 3: interferon regulatory factor 1 or 3, MHC: major histocompatibility complex, NFκB: nuclear factor κB, PD-(L)1: programmed death-ligand 1, STAT: signal transducer and activator of transcription, STING: stimulator of interferon genes, TBK-1: TANK-binding kinase 1, TLR4: Toll-like receptor 4, Trex1: three prime repair exonuclease 1.

Excitingly, in recent years, preclinical models exploring these novel, selective DDR inhibitors (DDRi) show that they have a surprising and potentially important effect: the ability to augment innate and/or adaptive anti-tumor immune responses. Here, we focus on the underlying mechanisms by which DDR pathway inhibition may augment anti-tumor immunity and potentially assist in overcoming resistance to ICI therapy (Figure 1). Combining an ATR inhibitor (ATRi) with an ICI, thereby targeting an essential regulator of cell cycle checkpoints in addition to immune checkpoint pathway blockade, may synergistically activate anti-tumor immunity while minimizing the toxicity of combined therapies.

**Figure 2:**
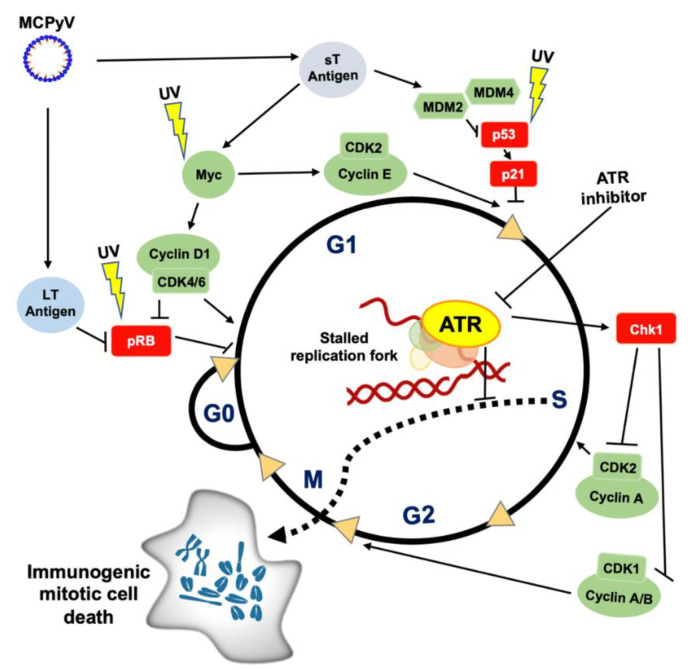
A model of how cell cycle dysregulation in Merkel cell carcinoma may predispose to ATR inhibition. Cellular components that restrain progression of the cell cycle are depicted in red; cell cycle progression accelerators are shown in green. In virus-positive (VP) MCC, Rb is directly inactivated by binding the MCPyV LT oncoprotein. p53 (via activation of MDM2/4) and Myc signaling are dysregulated in VP-MCC by the sT oncoprotein. Similarly, in virus-negative (VN) MCC, UV mutations disrupt Rb and p53 and promote Myc signaling. These changes disable the G1 cell cycle checkpoint in both VP- and VN-MCCs, making it potentially more reliant on ATR to ensure completion of DNA replication in S and G2 phases of the cell cycle. Stalled replication forks normally recruit ATR and lead to Chk1 activation. If ATR is inhibited, this disrupts the Chk1-dependent activation of the intra-S and G2/M checkpoints. This in-turn causes stalled replication forks to not be detected, may lead to double-strand breaks and, as depicted by the dashed arrow, promotes premature entry into M phase. This process may manifest as premature chromatin condensation (PCC) and lead to an immunogenic type of mitotic cell death. Abbreviations: CDK: cyclin dependent kinase, Chk1: checkpoint kinase 1, G0 phase: resting phase, G1 or G2 phase: Gap 1 or 2 phase, LT: Large T antigen, McPyV: Merkel cell polyomavirus, Mdm2 or 4: mouse double minute 2 or 4 homolog, M phase: mitosis phase, Myc: myelocytomatosis protein, PCC: premature chromatin condensation, p53: tumor protein p53, Rb: retinoblastoma protein, S phase: DNA synthesis phase, sT: small T antigen, UV: ultraviolet radiation.

There is substantial preclinical evidence relating targeted DDR pathway inhibition to anti-tumor immunity, as schematized in Figure 1 and described below: 

(1) With the recent availability of highly selective DDR pathway-targeting agents, inhibition of ATR, ATM and DNA-PK has been heavily explored preclinically, and to a lesser extent in clinical trials, to sensitize tumors to radiation and other DNA-damaging agents [67,87,88,89,90,91,92,93,94,95,96].

(2) Interestingly, recent studies demonstrate how DDR pathway signaling regulates the innate immune response by limiting micronuclei (MN) formation and cytosolic dsDNA accumulation [97,98,99,100,101]. In the presence of DDRi, however, cytosolic dsDNA leads to upregulated cGAS (cyclic GMP-AMP synthase)-STING (stimulator of interferon genes) signaling to induce type I interferon (IFN). Indeed, inhibition of ATR [102,103,104], ATM [105,106,107,108] or DNA-PK [109] activity results in enhanced innate immune responses in vitro and in mice.

(3) Following radiation-induced release of dsDNA into the cytosol, a DNA exonuclease, Trex1 (three prime repair exonuclease 1), suppresses anti-tumor immunity by degrading DNA and thus depriving cGAS-STING of its stimulatory signal [110,111]. Reduced expression of Trex1 in human cancer cell lines is associated with increased sensitivity to an ATRi [112]. 

(4) Radiation alone may upregulate MHC-I (major histocompatibility complex class I) expression and antigen presentation [113,114,115,116]. ATR, ATM and DNA-PK inhibition may also increase MHC-I expression in tumor cells to effect antigen presentation and T cell infiltration [102,107,117]. 

(5) Radiation upregulates PD-L1 on tumor cells which may affect their immunogenicity [45]. DDR pathway inhibition may also affect PD-L1 expression on tumor cells, although there is significant heterogeneity of the effect in different model systems. For example, ATR inhibition alleviated dsDNA break-induced PD-L1 upregulation in various cancer cell lines [118,119]. Similarly, in cultured HCC cells, ATRi treatment in combination with radiation decreased PD-L1 expression on tumor cells [120]. Conversely, targeted inhibition of Chk1 (checkpoint kinase 1, a critical effector kinase for ATR) induced PD-L1 expression in both human and murine small cell lung cancer cell lines [100]. Ionizing radiation-induced PD-L1 expression in U2OS cells was noted to be suppressed by an ATMi [119]. In human lung cancer cell lines, DNA-PK inhibition downregulated PD-L1 while increasing MHC-I expression [117] which could lead to improved activation of anti-tumor T cells. In summary, preclinical data suggest that DDR pathways often impinge on immune checkpoint biology, and this area warrants further study.

(6) ATR inhibition may selectively sensitize rapidly proliferating tumor cells via premature chromatin condensation (PCC) which can promote MN formation, subsequent innate immune signaling and potentially immunogenic cell death (ICD) [121,122,123,124,125] (see Figure 2 for details). 

(7) Radiation induces ICD through release of damage-associated molecular patterns (DAMPs) that activate dendritic cells (DCs) and thus augment the adaptive immune response [126]. Combining ATR inhibition with DNA-damaging agents may markedly increase the expression of DAMPs (surface-exposed calreticulin, high mobility group box protein 1 (HMGB1) and adenosine 5′-triphosphate (ATP) release) [127,128]. As depicted in Figure 1, DAMPs displayed by the tumor cell, HMGB1 and calreticulin, interact with their receptors on DCs, toll-like receptor 4 (TLR4) and CD91 (cluster of differentiation 91), respectively [129,130,131,132,133]. 

(8) and (9) Cytosolic DNA released from tumor cell-derived MN is transported within exosomes to DCs [134]. This induces type I IFN production via cGAS-STING activation in DCs [135,136,137]. ATR inhibition may enhance cGAS-STING signaling and DC activation in murine tumors to potentiate anti-tumoral cytotoxic T cells [102,138]. In addition to inducing ICD, combining an ATRi with RT may also induce senescence in a manner that could depend upon cGAS [139,140].

Several recent and exciting preclinical studies in immune-competent mouse models have explored the potential of DDR pathway inhibition to augment anti-tumor immunity. AZD6738 (ATRi) synergized with low-dose radiation to induce T cell-mediated tumor rejection in a mouse model of Kras-driven colorectal cancer [141]. Similarly, AZD6738 synergized with low-dose radiation to activate an IFN response, promote antigen presentation and increase T and natural killer (NK) cell infiltration, thereby improving tumor control in a murine model of HPV-driven cancer [102]. In a mouse model of HCC, the addition of an ATRi to radiation plus an anti-PD-L1 antibody increased anti-tumor CD8 T cell activity, enhanced immune memory and prolonged survival relative to radiation + ICI alone [120]. The observed efficacy of ATRi + ICI + radiation was mediated via cGAS-STING signaling [120]. Similarly, inhibition of Chk1 in a murine small cell lung cancer model potentiated ICI and was dependent on the cGAS-STING pathway [100]. Therefore, inhibition of ATR signaling augments innate immunity to damaged DNA in a variety of preclinical models, and an ATRi may augment ICI activity.

Loss of ATM or DNA-PK signaling may also increase the immunogenicity of tumors. Cells derived from patients with ATM deficiency and ATM-deficient mice both have increased type I IFN responses via the cGAS-STING pathway [105]. ATM inhibition (via small molecule or short-hairpin RNA) enhanced anti-tumor immunity in combination with ICI and radiation in murine models of ovarian and pancreatic cancers [107,108]. Inhibition (or genetic deficiency) of DNA-PK may similarly enhance innate immunity via cGAS-STING signaling [109]. DNA-PK mutations have been associated with a high TMB and high ICI response rates in several human cancers, and a DNA-PKi enhanced the efficacy of PD-1 pathway blockade in a murine colon cancer model [142]. In summary, there is substantial preclinical evidence that DDR pathway inhibition can augment innate and adaptive anti-tumor immunity. DDRi warrant further clinical study in the setting of ICI-refractory disease.

## 3. Clinical Experience with DNA Damage Response Pathway Inhibitors

Clinical testing of potent and selective inhibitors of DDR pathways has been underway for several years. The focus of this review is ATR, ATM and DNA-PK inhibitors, although PARPi have reached clinical practice, and early-phase trials of other DDRi have been conducted. Notably, Chk1 inhibitors were the first DDRi to undergo clinical trials and had unfavorable toxicity profiles [83,143]. Per review of clinicaltrials.gov in May 2021, greater than 80 trials in phases I or II are investigating ATRi, ATMi or DNA-PKi in a variety of settings, as reviewed elsewhere [82,144]. To briefly summarize the clinical landscape, there are five ATR inhibitors in clinical trials including AstraZeneca’s oral drug ceralasertib (AZD6738), with 33 trials listed, in addition to EMD Serono’s intravenous (IV) berzosertib (VX-970/M6620; 23 trials) and oral VX-803 (3 trials), Bayer’s oral BAY1895344 (8 trials) and Atrin Pharmaceuticals’ ATRN-119 (1 trial). Clinical publications describe early safety and efficacy data for ceralasertib [145], BAY1895344 [146] and berzosertib [147,148] in a variety of settings including as monotherapy or in combination with various DNA-damaging agents. ATRN-119 is a next-generation ATRi with enhanced potency and specificity that is entering early-phase trials [149]. Four ATM inhibitors, including the brain-penetrant, oral AZD1390 [150], are being studied in six trials. Five DNA-PK inhibitors are in early-phase trials. EMD Serono’s oral peposertib (M3814 [151]) is the most widely tested with 14 trials in a variety of settings and combinations with chemotherapies or radiotherapy. As summarized in Table 1, three ATR inhibitors (notably ceralasertib in combination with durvalumab in nine studies) and the DNA-PKi M3814 (with avelumab and radiation) are now being clinically tested in combination with ICIs. No clinical trials combining DDRi and ICI have yet to be completed and published to our understanding, al-though early meeting abstracts suggest the combinations are well tolerated.

## 4. Rationale for “Double-Checkpoint Inhibition” in Merkel Cell Carcinoma

The preclinical evidence for potential synergy between DDRi and ICIs is mounting. Investigation of DDR pathway inhibition in ICI-refractory disease is warranted, and multiple DDRi with favorable safety profiles are in early-phase clinical studies. For the reasons below, MCC may be an excellent test case to evaluate the ability of an ATRi to reinvigorate anti-tumor immunity in PD-1 pathway blockade-resistant disease.

MCC is an aggressive neuroendocrine cancer with two distinct etiologies. In the United States, ~80% of MCCs are caused by Merkel cell polyomavirus (MCPyV) whereby the viral T antigens dysregulate essential G1 (gap 1) cell cycle checkpoints to effectively transform the cell and drive unconstrained growth [152]. The remaining 20% of MCC cases result from UV-induced mutations affecting many of the same key G1 cell cycle checkpoint pathways as the MCPyV oncoproteins.

In virus-positive (VP) MCC, patient outcomes are correlated with the presence of CD8 T cells specific for viral protein antigens [153]. Both VP-MCC and virus-negative (VN) MCC are inherently immunogenic and are among the most responsive solid tumors to PD-1 pathway blockade, with approximately 50% of patients achieving durable disease control [25,154,155,156,157]. VN-MCC is characterized by an exceptionally high TMB, a well-known predictor of response to anti-PD-(L)1 therapy, as discussed above [16]. There is, however, a significant unmet medical need for MCC patients whose tumors have primary or acquired resistance to PD-1 pathway blockade. Given its characteristic immunogenicity, G1 cell cycle checkpoint deficiencies and associated sensitivity to DNA-damaging agents, we propose that MCC is a highly appealing tumor to test whether ATR inhibition can overcome resistance to PD-1 pathway blockade.

Both VP- and VN-MCC are driven by many of the major “PARCB” factors (p53, Akt1, RB1, c-Myc and Bcl2) of small cell neuroendocrine carcinogenesis [152,158,159]. As depicted in Figure 2, VP- and VN-MCCs are deficient in Rb and p53 signaling, while Myc (myelocytomatosis) signaling is upregulated. The viral Large T (LT) antigen interacts directly with Rb, a negative regulator of cell cycle progression through G1, to inhibit its activity [160]. p53 signaling is deficient in VP-MCC, although the gene is typically wild-type and the T antigens do not appear to directly inhibit its function [160,161]. Rather, small T (sT) antigen increases the activity of MDM2 (mouse double minute 2 homolog), a ubiquitin ligase regulating p53 activity, via activation of a transcriptional complex including L-Myc [162]. sT-mediated upregulation of L-Myc activity also promotes cell cycle progression through G1 [163]. Signaling via Rb [164,165], p53 [164,166] and Myc [167] is similarly dysfunctional in VN-MCC; however, this is via direct UV-induced mutations in these critical regulatory genes [152,168,169,170]. The net effect of deregulating the G1 cell cycle checkpoints is a highly proliferative malignancy, as evidenced by median Ki-67 positivity of >50% [171,172] relative to <5% Ki-67-positive tumor cells in malignant melanoma [173].

Loss of these early G1 cell cycle checkpoints renders cells more reliant upon the later S and G2/M cell cycle checkpoints, coordinated by ATR and its downstream effector kinase Chk1 [174,175], to ensure that DNA is replicated prior to entering mitosis (Figure 2). ssDNA at stalled replication forks in the S phase is sensed by ATR which is recruited to regions of ssDNA that have been coated with RPA (replication protein A) [176,177]. ATR subsequently activates Chk1 which signals downstream to halt cell cycle progression via inhibition of CDK2 (cyclin dependent kinase 2)/cyclin A in the S phase or in G2/M via inhibition of CDK1 paired with either cyclin B or cyclin A. As discussed above, complete loss of ATR function is not tolerated in replicating cells in contrast to ATM and DNA-PK which may be lost in human cancers. While ATR function is critical, low-dose pharmacologic inhibition of ATR may be tolerated by untransformed cells while selectively suppressing the growth of H-ras mutant and c-Myc-overexpressing fibroblasts, suggesting an increased reliance upon ATR in oncogene-expressing cells [178] (Figure 2). In the setting of an ATRi, cancer cells proceed prematurely to mitosis prior to completion of DNA synthesis in the S phase, which may manifest as PCC [121] and potentially an immunogenic type of mitotic cell death, as schematized in Figure 2 [125]. MCC tumors are highly replicative with the majority of cells actively dividing, as evidenced by high Ki-67. As such, MCC tumor cells are under significant replication stress and should be more dependent upon ATR to induce the DDR in S/G2 phases to prevent premature entry to mitosis and subsequent cell death. Therefore, ATR inhibition may allow cells to progress through the cell cycle with accumulated DNA damage (Figure 2) and potentiate ICD (Figure 1).

Rapidly dividing cells are particularly sensitive to DNA-damaging agents, as opposed to quiescent G0 cells which are resistant. MCC is sensitive to radiotherapy with an overall response rate of greater than 90% in metastatic lesions receiving a single 8 gray treatment [179]. Responses to radiation in immune-competent patients were durable (<10% in-field progression), whereas in-field tumor progression was observed in 30% of immunodeficient patients [179]. Radiation upregulates tumor cell antigen presentation via MHC-I, the downregulation of which is a well-established and dominant escape mechanism in immunogenic cancers including MCC [180,181]. MCC is notably chemotherapy-sensitive, although responses are short-lived [182,183,184]. Cytotoxic regimens are lymphodepleting and likely to antagonize the activity of an ICI. Targeting tumor cells with low-dose, conformal radiotherapy and an ATRi, which is selectively cytotoxic in rapidly dividing cells, may reduce the burden of MCC without suppressing systemic immunity which is critical for controlling MCC. Using radiotherapy, particularly hypofractionated regimens which are often considered more immunogenic [42,110], to effect DNA damage and potentiate ICI has been heavily explored over the past decade. The addition of an ATRi may help to potentiate the pro-inflammatory effects of radiation rather than its immunosuppressive properties [102].

## 5. Clinical Trials Including Patients with Merkel Cell Carcinoma

As summarized in Table 2, more than 80 clinical trials are potentially available for MCC patients or have been recently completed per review of clinicaltrials.gov in May 2021. As MCC is a rare cancer, the majority of these are early-phase studies open to patients with several advanced solid tumors. There are currently no approved therapies for ICI-refractory MCC, and these trials may provide important data to address this area of need. As discussed above, MCC is a highly immunogenic cancer, and, accordingly, novel immunotherapy-based treatments are well represented (Table 2) [185]. In addition to trials of novel ICIs, several classes of immunostimulatory agents are under study including T cell co-stimulatory molecules, intralesional therapies designed to activate innate immune receptors and oncolytic viruses. Adoptive T and NK cell-based strategies are being tested. Antagonists of immunosuppressive myeloid cells and associated signaling pathways are being explored [186,187,188]. MCC is notably sensitive to radiation, and several trial designs incorporate radiotherapy in various forms. Agents targeting cell cycle, cell death and cell proliferation pathways are also being tested and may be relevant given the biology of MCC reviewed above. While few of these trials are powered to assess efficacy, their related translational analyses and exploratory endpoints may yield insight into the development of new therapies for ICI-resistant MCC.

## 6. Discussion and Conclusions

Beginning with the initial approval of ipilimumab for malignant melanoma in 2011, highly immunogenic skin cancers have been important model diseases for the development of new immune-directed therapies. ICIs have been transformational for a subset of patients with advanced skin cancers and have provided us with unique insights into the importance of UV neoantigens and viral antigens in anti-tumor immunity. However, several recent immune-focused approaches have not meaningfully improved upon the early gains from targeting the CTLA-4 and PD-(L)1 pathways. While incremental improvements from new and combined immune therapies are also anticipated, it may be important to target processes beyond the immune system to augment the efficacy of ICIs. Such orthogonal approaches to enhancing immunity may provide a synergistic therapeutic benefit while avoiding excess immune toxicity. Skin cancer, particularly MCC, may again prove a fertile testing ground for understanding whether DDRi can augment immunity and potentiate existing immunotherapies. Exciting new biology and the availability of specific small molecules inhibiting central DDR pathway regulators including ATR, ATM and DNA-PK facilitate clinical trials of DDRi with a focus on enhancing anti-tumor immunity.

**Figure 3:**
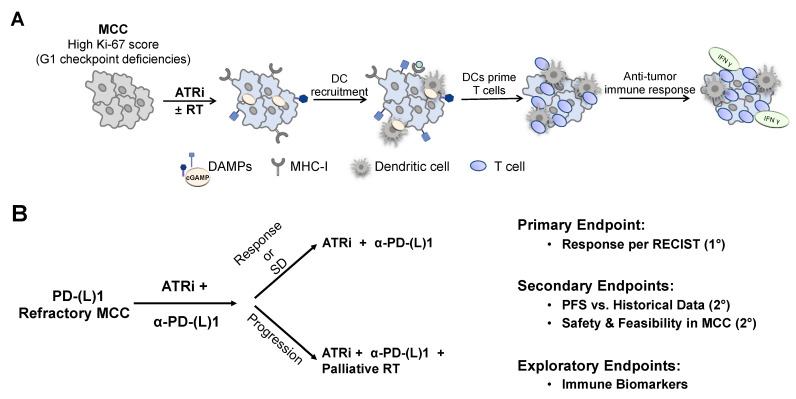
Potential role for an ATR inhibitor (ATRi) to overcome immune checkpoint inhibitor (ICI)-refractory Merkel cell carcinoma (MCC) and a clinical trial concept. (**A**) Prospective mechanism of action for an enhanced immune response in MCC treated with an ATRi ± radiotherapy (RT). (**B**) Schematic for a potential clinical trial of an ATRi for patients with MCC refractory to ICIs wherein an ATRi would be added to a patient’s ongoing PD-1 pathway blockade. Palliative RT may be utilized to control progressive lesions and potentially synergize with the ATRi. Abbreviations: cGAMP: cyclic guanosine monophosphate–adenosine monophosphate, α-PD-(L)1: anti-PD-(L)1, PFS: progression-free survival, RECIST: response evaluation criteria in solid tumors, SD: stable disease.

In addition to improving survival, maintaining the quality of life for those with advanced disease is a central goal for our patients. Widening the therapeutic window may be achievable by combining therapies with distinct cellular targets and synergistic efficacy but without known overlapping toxicities. A DDR pathway inhibitor paired with an ICI, targeting cell cycle and immune checkpoints, respectively, is one such promising combination. DDRi may slow tumor growth by restricting cell cycle progression while also promoting immune signaling and ICD to potentiate innate and systemic anti-tumor immunity (Figure 3A). The addition of very low dose radiation (potentiated by the tumor-selective radiosensitizing properties of an ATRi) may lower the tumor burden to facilitate the immune system’s ability to control microscopic disease and is expected to be well tolerated.

The rationale for a study of an ATRi in advanced MCC is driven not only by the potential to restrict tumor growth via direct effects on cell cycle progression but also by the mounting preclinical evidence for ATR inhibition to promote anti-tumor immunity in this remarkably ICI-sensitive cancer. In particular, a clinical trial is envisioned for patients with ICI-refractory MCC in which an ATRi would be added while continuing PD-1 pathway blockade (Figure 3B). Such a study would assess the efficacy of an ATRi to stabilize metastatic disease and re-establish anti-tumor immunity. Targeted, low-dose radiotherapy may be employed to palliate progressive lesions and potentially synergize with “double-checkpoint inhibition”. Correlative studies should address how an ATRi may modulate innate and adaptive anti-tumor responses and what the mechanisms of resistance to an ATRi might be.

DDR pathway inhibitors with favorable toxicity profiles are now in phase I and II clinical trials. Preclinical data supporting a role for DDR pathway inhibition to promote innate and systemic anti-tumor immunity provide a strong rationale for clinical trials combining DDRi with immune-targeted therapies to address ICI-resistant disease. Excitingly, ceralasertib (AZD6738), an ATRi, in combination with durvalumab showed promising efficacy (overall response rate of 30% and disease control rate of 63%) in patients with melanoma resistant to anti-PD-1 therapy per an early report at ASCO in 2021 [189]. These data are encouraging when considering the potential benefit of an ATRi plus anti-PD-(L)1 therapy in MCC patients with ICI-resistant disease. Additionally, strong correlative studies to assess biomarkers of response and resistance are critical to further our understanding of the interplay between DDR pathways and the immune system in human disease.

## Figures and Tables

**Table 1:** Clinical trials pairing DNA damage response inhibitors (DDRi) with immune checkpoint inhibitors (ICIs). The inhibitory concentration (IC_50_) indicates the potency of the inhibitor. ATM is not listed because no ICI combinations with ATM were listed on clinicaltrials.gov at the time of review.

DDRi Agent	Chemical Structure, IC_50_	Total No. of Trials	Trials with ICI	Phase	ICI Agents in Combination with DDRi
**ATR inhibitors**					
AZD6738/ Ceralasertib Oral, AstraZeneca	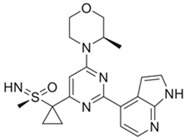 Cell-based IC_50_ = 74 nM	33	9	I/II, II	Durvalumab, Cisplatin/Carboplatin + Etoposide + Durvalumab
VX-970/M6620/ Berzosertib Intravenous, EMD Serono	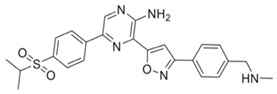 Cell-based IC_50_ = 19 nM	23	3	I, I/II	Avelumab, Avelumab + Carboplatin, Carboplatin + Gemcitabine hydrochloride + Pembrolizumab
BAY1895344/ Elimusertib Oral, Bayer	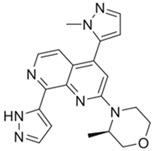 Cell-based IC_50_ = 36 nM	8	2	I	Pembrolizumab, Stereotactic body radiation therapy + Pembrolizumab
**DNA-PK inhibitor**					
M3814/MSC2490484A/ Nedisertib/ Peposertib Oral, EMD Serono	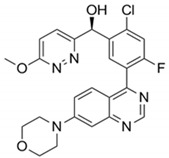 Cell-based IC_50_ < 0.5 μM	14	3	I, I/II	Avelumab ± radiotherapy, Avelumab + Radium-223 Dichloride, Avelumab + Hypo-fractionated radiotherapy

**Table 2:** Novel therapeutic agents potentially available to MCC patients on clinical trials or with recently completed trials.

Drug Class	Sub-Class	Specific Agents	Phase
Immune checkpoint inhibitors (ICI)	Combination ICI	Ipilimumab (anti-CTLA-4) + Nivolumab (anti-PD-1)	I, II
	Novel ICI	INCAGN02385 (Lag3), INCAGN02390 (Tim3)	I
	New PD-(L)1/CTLA4 mAbs	BT-001 (Treg depleting anti-CTLA-4), Tremelimumab (anti-CTLA-4), retifanlimab (anti-PD1)	I/II, II
Radiotherapy	Radiotherapy + ICI	Ipilimumab/Nivolumab + SBRT; Pembrolizumab + SBRT	II
	Novel radiosensitizer	NBTXR3 (radioenhancer hafnium oxide nanoparticle)	I
Immune agonists	T cell co-stimulatory agonist	INCAGN01949 (OX40), INCAGN01876 (GITR), Utomilumab (4-1BB)	I, I/II
	Intralesional innate immune agonists	Poly-ICLC (TLR3), Imiquimod (TLR7), NKTR-262 (TLR7/8), Cavrotolimod (TLR9)	I, I/II
	Oncolytic viruses	T-VEC (Herpes virus), Ad-p53 (adenovirus expressing p53)	I, II
	Tumor vaccines	IFx-Hu2.0 (DNA vaccine with streptococcal antigen)	I
	Cytokines	Bempegaldesleukin (CD122-preferential IL-2 pathway agonist), NT-I7 (IL-7 agonist), N-803 (IL-15 superagonist +ICI or NK cells), SO-C101 (IL-15 superagonist)	I, I/II, II
Adoptive cell therapies	T cells	MCPyV T antigen-specific polyclonal autologous CD8+ T cells	I/II
	NK cells	Allogeneic NK cell	Case only
Novel agents targeting immunosuppressive TME	NOS inhibitor	L-N^G^-monomethyl Arginine acetate (L-NMMA)	I
	Adenosine antagonist	Etrumadenant	I
	IDO1 inhibitor	Epacadostat	I/II
Tumor antigen targeted therapy	Somatostatin	Lanreotide (SST analogue), Tidutamab/XmAb18087 (bispecific mAb targeting CD3 and SSTR)	I/II, II
	Targeted radionuclide	Lutetium-177 DOTATATE (targeting SSTR)	I/II
Antibody–drug conjugates	Anti-DLL3	Rovalpituzumab tesirine (anti-DLL3 with DNA cross-linking drug)	I
	Anti-CD56	huN901-DM1 (anti-CD56 with microtubule inhibitor)	I
Cell cycle/cell death and proliferation pathways	MDM2 inhibitor	KRT-232	II
	Anti-Bcl2	Oblimersen	II
	mTOR inhibitors	RAD001, MLN0128	I, I/II
Anti-angiogenics	Anti-VEGF mAb	Bevazicumab (with atezolizumab)	I/II, II
	Tyrosine kinase inhibitor	Vatalanib (small molecule targeting VEGF receptors, PDGF receptor beta and c-kit)	I

Abbreviations: 4-1BB: TNF receptor family co-stimulatory receptor, also known as CD137 and TNFRS9; Ad-p53: oncolytic adenovirus transgenically expressing p53; Bcl2: B cell lymphoma 2; CD 3/8/56/122: cluster of differentiation 3/8/56/122; CTLA-4: cytotoxic T-lymphocyte-associated protein 4; DLL3: delta-like protein 3; GITR: glucocorticoid-induced TNFR-related protein; ICI: immune checkpoint inhibitors; IDO1: indoleamine 2,3-dioxygenase; IL-2/7/15: interleukin-2/7/15; L-NMMA: L-NG-monomethyl arginine acetate; Lag3: lymphocyte activating 3; mAb: monoclonal antibody; MCPyV: Merkel cell polyomavirus; MDM2: mouse double minute 2 homolog; mTOR: mammalian target of rapamycin; NK cell: natural killer cell; NOS: nitric oxide synthase; OX40: tumor necrosis factor receptor superfamily, member 4, also known as CD134 and OX40 receptor; PD-1: programmed cell death-1; PDGF: platelet-derived growth factor; PD-(L)1: programmed cell death-1 ligand 1; SBRT: stereotactic body radiation therapy; SST: somatostatin; SSTR: somatostatin receptor; T-VEC: talimogene laherparepvec; Tim3: T cell immunoglobulin and mucin domain-containing protein 3; TLR3/7/8/9: Toll-like receptor 3/7/8/9; TME: tumor microenvironment; VEGF: vascular endothelial growth factor.

## Data Availability

Not applicable.
